# Does Seasonal Influenza Vaccination Increase the Risk of Illness with the 2009 A/H1N1 Pandemic Virus?

**DOI:** 10.1371/journal.pmed.1000259

**Published:** 2010-04-06

**Authors:** Cécile Viboud, Lone Simonsen

**Affiliations:** 1 Division of Epidemiology and Population Studies, Fogarty International Center, National Institutes of Health, Bethesda, Maryland, United States of America; 2 Department of Global Health, School of Public Health and Health Services, George Washington University, Washington, D. C., United States of America

**Linked Research Article**

This Perspective discusses the following study published in *PLoS Medicine*:

Skowronski DM, De Serres G, Crocroft N, Janjua NZ, Boulianne N, et al. (2010) Association between the 2008–09 Seasonal Influenza Vaccine and Pandemic H1N1 Illness during Spring–Summer 2009: Four Observational Studies from Canada. PLoS Med 7(4): e1000258. doi:10.1371/journal.pmed.1000258

In three case-control studies and a household transmission cohort, Danuta Skowronski and colleagues find an association between prior seasonal flu vaccination and increased risk of 2009 pandemic H1N1 flu.

## Background

As the novel pandemic influenza A (H1N1) (pH1N1) virus spread around the world in late spring 2009 with a well-matched pandemic vaccine not immediately available, the question of partial protection afforded by seasonal influenza vaccine arose. Coverage of the seasonal influenza vaccine had reached 30%–40% in the general population in 2008–09 in the US and Canada, following recent expansion of vaccine recommendations.

Serology studies demonstrated a lack of cross-reactive antibody to the novel virus in vaccinated and unvaccinated people under 60 years of age, suggesting that there would be no protection against pandemic influenza from natural immunity or seasonal vaccination [1]. By contrast, about one third of seniors over 60 y had cross-reactive antibodies [1], perhaps due to childhood exposure to antigenically similar A/H1N1 viruses. As a result, the mean age of pandemic cases and deaths was younger than that of interpandemic seasons [2], a signature age shift also experienced in three historical influenza pandemics [3].

## Unexpected Findings in a Sentinel Surveillance System

The spring 2009 pandemic wave was the perfect opportunity to address the association between seasonal trivalent inactivated influenza vaccine (TIV) and risk of pandemic illness. In this issue of *PLoS Medicine*, Danuta Skowronski and colleagues report the unexpected results of a series of Canadian epidemiological studies suggesting a counterproductive effect of the vaccine [4]. The findings are based on Canada's unique near-real-time sentinel system for monitoring influenza vaccine effectiveness. Patients with influenza-like illness who presented to a network of participating physicians were tested for influenza virus by RT-PCR, and information on demographics, clinical outcomes, and vaccine status was collected. In this sentinel system, vaccine effectiveness may be measured by comparing vaccination status among influenza-positive “case” patients with influenza-negative “control” patients. This approach has produced accurate measures of vaccine effectiveness for TIV in the past, with estimates of protection in healthy adults higher when the vaccine is well-matched with circulating influenza strains and lower for mismatched seasons [5]. The sentinel system was expanded to continue during April to July 2009, as the pH1N1 virus defied influenza seasonality and rapidly became dominant over seasonal influenza viruses in Canada.

## Additional Analyses and Proposed Biological Mechanisms

The Canadian sentinel study showed that receipt of TIV in the previous season (autumn 2008) appeared to increase the risk of pH1N1 illness by 1.03- to 2.74-fold, even after adjustment for comorbidities, age, and geography [4]. The investigators were prudent and conducted multiple sensitivity analyses to attempt to explain their perplexing findings. Importantly, TIV remained protective against seasonal influenza viruses circulating in April through May 2009, with an effectiveness estimated at 56% (41%–67%), suggesting that the system had not suddenly become flawed. TIV appeared as a risk factor in people under 50 y, but not in seniors—although senior estimates were imprecise due to lower rates of pandemic illness in that age group. Interestingly, if vaccine were truly a risk factor in younger adults, seniors may have fared better because their immune response to vaccination is less rigorous [6].

Because of the potential public health seriousness of the findings, complementary observational studies were launched in Ontario and Quebec, based on hospital and community cases and controls. These studies confirmed TIV as a risk factor for 2009 pH1N1 illness, but were somewhat reassuring in that TIV did not increase severity of disease. Finally, a household study in Quebec did not show a convincing difference in secondary attack rates by vaccination status, although the statistical power was rather limited.

The authors proposed several biological mechanisms to explain why seasonal vaccination may increase the risk of pandemic illness [4]. One mechanism involves lack of heterosubtypic immunity in recipients of TIV, as heterobsutypic immunity may be generated through T cell responses during natural infection with seasonal influenza viruses, but not through vaccination. This explanation remains hypothetical, as biological evidence of heterosubtypic immunity in humans is scarce despite circumstantial evidence from past pandemics [7],[8]. Other proposed mechanisms were original antigenic sin and antibody-dependent enhancement, whereby TIV may induce high antibody titers to seasonal influenza viruses, which may cross-react with pH1N1 without neutralizing it, and counteract development of a robust antibody response to pandemic influenza infection. However, the evidence that antibody response in human populations depends on the sequence of past influenza infections remains debated. Overall, full characterization of baseline pre-pandemic immune profiles of recipients of inactivated and live-attenuated seasonal influenza vaccines and of unvaccinated individuals of various ages, would be highly informative to basic science and public health. Hopefully, such key studies can still be conducted in part by analysis of stored blood bank sera.

## Potential Biases and Findings from Other Countries

The Canadian authors quickly found themselves at odds with expert review committees who were not convinced by the data and largely dismissed the findings as due to confounding bias—a fair criticism of observational studies. To their credit, the authors had thoroughly assessed potential biases in their article [4], in particular relative to the selection of controls and differences in health care–seeking behavior, and repeated the study in different Canadian provinces. They also provided a full description of their study population and carefully compared vaccine coverage and prevalence of comorbidities in controls with national or province-level age-specific estimates—the best one can do short of a randomized study. In parallel, profound bias in observational studies of vaccine effectiveness does exist, as was amply documented in several cohort studies overestimating the mortality benefits of seasonal influenza vaccination in seniors [9].

Given the uncertainty associated with observational studies, we believe it would be premature to conclude that TIV increased the risk of 2009 pandemic illness, especially in light of six other contemporaneous observational studies in civilian populations that have produced highly conflicting results (see Table 1 for details on study design, population sampled, and results) [10]–[15]. We note the large spread of vaccine effectiveness estimates in those studies; indeed, four of the studies set in the US and Australia did not show any association [12]–[15], whereas two Mexican studies suggested a protective effect of 35%–73% [10],[11]. The most recent Canadian study in this issue of *PLoS Medicine* [4] is clearly at odds with these results, with an estimated average negative effectiveness of −68% based on their Sentinel system. Only one study, set in the US military population, potentially corroborated the findings of the Canadian study [16].

**Table 1:** Comparison of observational studies evaluating the effectiveness of seasonal influenza vaccination to prevent 2009 pA/H1N1 morbidity in civilian populations.

Reference	Study Setting	Study Design	Sample Size (% of Young Adults)	Outcome	Vaccine Effectiveness Estimate^a^ (95% CI)
Skowronski et al. [5]	Canada; 17 April to 22 July 2009 (first wave)	Sentinel test-negative case control; community-based GPs^b^	144 cases, 536 controls (49% aged 20–49 y)	2009 H1N1pdm RT-PCR-confirmed	−68% (−174% to −3%)b, c
Garcia-Garcia et al. [10]	Specialty hospital, Mexico City, Mexico; 29 March to 20 May 2009	Matched hospital case-control	60 cases; 180 controls (63% aged 21–60 y)	2009 H1N1pdm RT-PCR-confirmed	73% (34% to 89%)^d^
Echevarria-Zuna et al. [11]	Mexico, 28 April to 31 July 2009	Prospective surveillance (case-negative controls in inpatients and outpatients)	1,766 cases; 8,096 controls (N/A)	2009 H1N1pdm RT-PCR-confirmed	35% (23% to 45%)
Kelly et al., [12]	Victoria state, Australia; 27 April to 12 July 2009	Sentinel test-negative case control; community-based GPs	212 cases; 365 controls (54% aged 20–49 y)	2009 H1N1pdm RT-PCR-confirmed	3% (−56% to 40%)^e^
Gargiullo et al., [13]	Eight states, USA; May–June 2009	Case-based (ie, case- cohort)	356 cases; vacc coverage sample size = 20,689 (77% aged 18–49 y)	2009 H1N1pdm RT-PCR-confirmed	−10% (−46% to 15%)^d^
Iuliano et al., [14]	University of Delaware outbreak, USA; 27 March to 9 May 2009	Online retrospective survey	7,450 respondents (90% aged 18–49 y); 677 had ILI	ILI (fever, sore throat or cough)	−10% (−40% to 10%)^d^
Lessler et al., [15]	New York city school outbreak, USA; April 2009	Online retrospective survey	2,225 respondents (0% over age 20 y); 694 had ILI	ILI (fever, sore throat or cough)	−5% (−20% to 9%)

All studies were conducted during the first wave of the 2009 pandemic (April–July 2009) and most include a majority of young adults.

a Negative estimates of vaccine effectiveness indicate that vaccination may be a risk factor for 2009 pandemic illness, while positive estimates suggest a protective effect.

b Three other study designs are considered in this publication but the Sentinel system is the most well-established.

c Adjusted for age, comorbidities, province, interval between symptoms onset and sample collection.

d Adjusted for age and comorbidities.

e Adjusted for age.

ILI, influenza like illness.

All studies, including [4], are potentially prone to bias due to lack of randomization. Perhaps the more extreme Canadian, US, and Mexican studies were deeply biased, or perhaps the population experiences were truly different due to their vaccination histories and past influenza exposure. Given the sudden spread of the pandemic virus, it would have been extremely difficult to design a prospective (randomized) trial to evaluate TIV effectiveness—and such a study is now forever complicated by pandemic vaccination efforts.

## Policy Implications and a Way Forward

The putative association between seasonal vaccination and 2009 pH1N1 illness remains an open question, given the conflicting evidence from available research. Canadian health authorities debated whether to postpone seasonal vaccination in the autumn of 2009 until after a second pandemic wave had occurred, but decided to follow normal vaccine recommendations instead, in part because of uncertainty about a resurgence of seasonal influenza viruses during the 2009–10 season [17]. This illustrates the difficulty of making policy decisions in the midst of a public health crisis, when officials must rely on limited and possibly biased evidence from observational data, even in the best possible scenario of a well-established sentinel monitoring system already in place.

What happens next? Given the timeliness of the Canadian sentinel system, data on the association between seasonal TIV and risk of pH1N1 illness during the autumn 2009 pandemic wave will become available very soon, and will be crucial in confirming or refuting the earlier Canadian results. In addition, evidence may be gained from disease patterns during the autumn 2009 pandemic wave in other countries and from immunological studies characterizing the baseline immunological status of vaccinated and unvaccinated populations. Overall, this perplexing experience should teach us how to best react to disparate and conflicting studies and prepare us for the next public health crisis, so that we can better manage future alerts for unexpected risk factors.

## Abbreviations

pH1N1: pandemic influenza A (H1N1)
TIV: trivalent inactivated influenza vaccine
